# Comorbid Learning Difficulties in Reading and Mathematics: The Role of Intelligence and In-Class Attentive Behavior

**DOI:** 10.3389/fpsyg.2020.572099

**Published:** 2020-11-16

**Authors:** David C. Geary, Mary K. Hoard, Lara Nugent, Zehra E. Ünal, John E. Scofield

**Affiliations:** Department of Psychological Sciences, Interdisciplinary Neuroscience, University of Missouri, Columbia, MO, United States

**Keywords:** learning difficulties, adolescence, reading achievement, mathematics achievement, cognition, attention, learning, memory

## Abstract

The goal was to identify the domain-general cognitive abilities and academic attitudes that are common and unique to reading and mathematics learning difficulties that in turn will have implications for intervention development. Across seventh and eighth grade, 315 (155 boys) adolescents (*M* age = 12.75 years) were administered intelligence, verbal short-term and working memory, and visuospatial memory, attention, and ability measures, along with measures of English and mathematics attitudes and mathematics anxiety. Teachers reported on students’ in-class attentive behavior. A combination of Bayesian and multi-level models revealed that intelligence and in-class attentive behavior were common predictors of reading accuracy, reading fluency, and mathematics achievement. Verbal short-term memory was more critical for reading accuracy and fluency, whereas spatial ability and mathematics self-efficacy were more critical for mathematics achievement. The combination of intelligence and in-class attentive behavior discriminated typically achieving students from students with comorbid (*D* = 2.44) or mathematics (*D* = 1.59) learning difficulties, whereas intelligence, visuospatial attention, and verbal short-term memory discriminated typically achieving students from students with reading disability (*D* = 1.08). The combination of in-class attentive behavior, verbal short-term memory, and mathematics self-efficacy discriminated students with mathematics difficulties from their peers with reading difficulties (*D* = 1.16). Given the consistent importance of in-class attentive behavior, we conducted *post hoc* follow-up analyses. The results suggested that students with poor in-class attentive behavior were disengaging from academic learning which in turn contributed to their risk of learning difficulties.

## Introduction

Academic competencies at the end of secondary school contribute to individuals’ employability, wages, and the ability to pursue further education (Rivera-Batiz, 1992; Bynner, 1997; Ritchie and Bates, 2013; Stoet and Geary, 2020). Individuals with deficits in core academic domains, especially reading and mathematics, will face long-term hardships in many areas of life (Richmond-Rakerd et al., 2020). Interventions that reduce risk of academic difficulties thus have the potential for long-term benefits for at-risk individuals and the communities in which they will eventually reside. Fuchs et al. (2013, 2019) demonstrated that students’ responsiveness to such interventions is influenced by their preexisting domain-general abilities, such as working memory, and that more effective interventions can be developed with the inclusion of supports that address any domain-general weaknesses (Fuchs et al., 2020). As an example, speeded (timed) practice of just-learned number knowledge benefited students with weak non-verbal reasoning abilities, whereas non-speeded practice did not.

More generally, individual differences in academic achievement, achievement growth, and grade-point average are related to domain-general cognitive abilities (e.g., working memory; Geary et al., 2017; Peng et al., 2019) and to non-cognitive factors, such as mathematics self-efficacy (Marsh and Yeung, 1998; Eccles and Wang, 2016; Semeraro et al., 2020). However, most of the learning difficulties research has focused on cognitive factors, such as poor working memory (Geary, 1993, 2004; Swanson et al., 2009a; Peng and Fuchs, 2016; Koponen et al., 2017), although non-cognitive factors are sometimes considered (Cirino et al., 2018; Devine et al., 2018). Even fewer studies have assessed the joint relations between domain-general cognitive abilities and non-cognitive factors and learning difficulties. We provided such an assessment for middle-school students and sought to determine the best combinations of domain-general cognitive and non-cognitive factors that characterize learning difficulties in mathematics, reading, and their comorbidity.

### Learning Difficulties

In the United States, about 5% of students have school-identified specific learning disabilities (Grigorenko et al., 2020), but the percentage of at-risk students is much higher. This is because many students fail to achieve grade-level learning benchmarks for key subject areas. The U.S. National Assessment of Educational Progress, for instance, identifies ‘basic’ achievement levels as *partial* mastery of grade-level knowledge and skills.^1^ The most recent assessments revealed that 27% and 28% of United States students were below basic levels of achievement in reading in 8th and 12th grade, respectively. For mathematics, 31% and 38% of students were below basic levels of achievement in 8th and 12th grade, respectively (United States Center for Educational Statistics, 2020).

Many students with below-basic academic competencies are not identified as having a specific learning disability and many of them might not meet the multiple criteria for diagnosis of such a disability (Grigorenko et al., 2020). Nevertheless, students with below basic levels of reading and mathematical competencies are at high risk of long-term difficulties in the labor market and in other areas of life (Berlin and Sum, 1988; Rivera-Batiz, 1992; Hanushek and Woessmann, 2008; Ritchie and Bates, 2013). In the study of the factors contributing to learning difficulties in mathematics, a commonly used cutoff is at or below the 25th percentile on a standardized achievement test (Geary et al., 2007; Murphy et al., 2007). A cutoff at this percentile is consistent with the percentage of United States students with below-basic academic competencies, and thus we adopted it for our analyses of reading, mathematics, and comorbid learning difficulties.

However, performance on achievement tests is continuous and generally normally distributed (Geary et al., 2012; Grigorenko et al., 2020), and thus cutoffs at any percentile are arbitrary, albeit useful for the identification and study of at-risk students. For this reason, we also use an individual differences approach to identify the domain-general cognitive and non-cognitive factors that are common and unique to reading and mathematics achievement.

### Domain-General Cognitive Abilities

Individual differences in reading and mathematics achievement, as well as achievement growth, are related to intelligence and executive functions (Deary et al., 2007; Peng et al., 2018, 2019). Working memory–holding information in mind while engaged in other processes (Miyake et al., 2000)–is an important component of executive functions and is consistently related to academic learning (Paas and Ayres, 2014; Lee and Bull, 2016; Geary et al., 2017). Although the diagnosis of a specific learning disability typically involves the exclusion of students with very low IQ scores (Grigorenko et al., 2020), students with persistent learning difficulties often have modestly lower IQ scores than their typically achieving peers. Although IQ may contribute to their learning difficulties, it is not in and of itself a sufficient explanation (Stuebing et al., 2002; Murphy et al., 2007). Deficits in executive functions and especially working memory appear to be an additional contributing factor in both reading difficulties and mathematics difficulties (Swanson et al., 2009b; Geary et al., 2012).

There are also cognitive abilities that are relatively more important for reading or for mathematics achievement. Short-term verbal memory and verbal working memory contribute to various aspects of reading competence–assessed in individual differences and learning disability studies–and are more important than visuospatial memory (Carretti et al., 2009; Swanson et al., 2009b; Giofrè et al., 2018; Peng et al., 2018). Verbal short-term and working memory can contribute to some aspects of early number and arithmetic learning (Krajewski and Schneider, 2009; Allen et al., 2020), but these become relatively less important for mathematics learning in later grades. As the mathematics that students are expected to learn becomes more complex, visuospatial memory (Li and Geary, 2013, 2017) and more complex spatial abilities become increasingly important (Casey et al., 1997; Kyttälä and Lehto, 2008). The latter includes the ability to generate and manipulate visual images and is consistently correlated with mathematics achievement (Casey et al., 1997). The ability to control visuospatial attention is related to some aspects of number processing (Longo and Lourenco, 2007) and may also contribute to word reading (Friedrich et al., 1985).

### Non-cognitive

Several non-cognitive measures were considered in this study, including academic attitudes, mathematics anxiety, and in-class attentive behavior. The attitudes included self-efficacy or confidence about one’s abilities in English and mathematics, as well as beliefs about the future usefulness or utility of competence in English and mathematics (Eccles and Wigfield, 2002; Eccles and Wang, 2016). The relation between these attitudes and academic outcomes is typically bidirectional for older students and adults (Valentine et al., 2004; Talsma et al., 2018), but the relations are less certain across elementary and middle school students (Giofrè et al., 2017; Gunderson et al., 2018; Geary et al., 2019; Toste et al., 2020). Whatever the direction of the relations during schooling, in the long-term these attitudes can influence later occupational choices (Lauermann et al., 2017).

Mathematics anxiety is another factor that has been linked to variation in mathematics outcomes, although cause-and-effect relations are not yet fully understood (Ashcraft and Kirk, 2001; Ma and Xu, 2004; Dowker et al., 2016); unfortunately, we did not have a parallel measure for reading anxiety. Byrnes and Miller-Cotto (2016) recently found that internalizing problems, which includes anxiety, were associated with slower mathematics growth across the third- and eighth-grade academic years, controlling many other factors. Higher mathematics anxiety is also thought to result in an avoidance of mathematics coursework and math-intensive careers (Hembree, 1990; Meece et al., 1990).

On the basis of these findings, we might expect that students with learning difficulties would show lower academic self-efficacy and those with mathematics difficulties would show higher mathematics anxiety than their typically achieving peers, but this is not always the case. The academic self-efficacy of many students with learning difficulties (in any area) is overly optimistic relative to their actual achievement (Klassen, 2002). Devine et al. (2018) found that relative to typically achieving students, about twice as many students with mathematics learning difficulties showed high levels of mathematics anxiety. However, most of the students with difficulties did not show higher than average levels of mathematics anxiety and many of the anxious students had average or better mathematics achievement levels.

A final non-cognitive factor that contributes to academic achievement is in-class attention. Teacher ratings of students’ in-class attentive behavior is consistently related to concurrent and longitudinal gains in mathematical achievement and is sometimes related to gains in reading achievement (Fuchs et al., 2006, 2016; Geary et al., 2013). The associated behaviors include sustained attention and attention to details during school activities, and distractibility in the classroom (Swanson et al., 2012). In-class attentive behavior is likely influenced by cognitive competencies, such as executive functions, but we included it as a non-cognitive measure because it captures aspects of students’ behavioral engagement in the classroom that are not fully captured by cognitive measures of attentional control and working memory.

Attentional deficits and issues with behavioral self-control are common among students with reading, mathematics, and comorbid learning difficulties (Willcutt et al., 2013), and have long-term consequences. In a large-scale longitudinal study, Smart et al. (2017) found that the combination of learning difficulties and comorbid attentional and behavioral deficits resulted in a 16-fold increase in the odds of dropping out of school and a 2-fold increase in the odds of employment difficulties in early adulthood.

### Current Study

The current study provides a broad assessment of the domain-general cognitive, as well as the non-cognitive factors, that are common and unique to individual differences in reading and mathematics achievement and in the prediction of comorbid learning difficulties. As noted, identifying the domain-general cognitive abilities that contribute to mathematics and reading achievement will have implications for the development of interventions for students who are at-risk for academic difficulties (Fuchs et al., 2013, 2019, 2020). The inclusion of non-cognitive factors greatly broadens the study of these at-risk students and could have implications for understanding their long-term engagement in the domain. For instance, above-average levels of mathematics anxiety could result in an avoidance of mathematics that over time will exacerbate knowledge-deficits in this domain (Hembree, 1990; Meece et al., 1990).

On the basis of prior studies, we anticipated that IQ and one or several measures of working memory (e.g., N-back) would emerge as common contributors to reading and mathematics achievement, and to comorbid learning difficulties. We also anticipated that one or several of the verbal short-term or working memory measures would be unique (or at least relatively more important) to reading achievement and difficulties, and that one or several of the visuospatial measures would be unique to mathematics achievement and difficulties. Among the non-cognitive measures, we anticipated in-class attentive behavior would emerge as important to mathematics and perhaps reading achievement but were less certain about the attitudes and anxiety measures, given the mixed findings in prior studies.

## Materials and Methods

### Participants

The participants were 315 (155 boys) students enrolled in an on-going longitudinal study conducted in collaboration with the Columbia Public Schools in Columbia, MO, United States. They were recruited across two cohorts from a larger group of 1,926 students who participated in an assessment of sixth-grade mathematical competencies (see Geary et al., 2019). All 1,926 students were invited to join the longitudinal component of the study and 342 of them and their parents did so. The 315 students included here completed all of the seventh- and eighth-grade assessment sessions.

Demographic information was obtained through a parent survey. For the group of 315 students, 88% of them were non-Hispanic, 6% were Hispanic or Latino, and the ethnic status of the remaining students was unknown. The racial composition was 71% White, 14% Black, 3% Asian, 1% Native American, 10% multiracial, with the remaining unknown. As a comparison, students in the school district from which the participants were recruited were 61% white, 20% black, 5% Asian, 7% Hispanic, and 6% multiracial. For the current participants, parent-reported annual household income was distributed as follows: $0–$24,999 (9%); $25,000–$49,999 (15%); $50,000–$74,999 (9%); $75,000–$99,999 (19%); $100,000–$149,999 (17%); and $150,000+ (15%). Sixty-three percent of the students had at least one parent with a college degree. Fifteen percent of the families received food assistance, and five percent received housing assistance.

### Materials

#### Standardized Measures

##### Intelligence

Full scale IQ was estimated using the Vocabulary and Matrix Reasoning subtests of the *Wechsler Abbreviated Scale of Intelligence* (WASI; Wechsler, 1999), following procedures detailed in the manual.

##### Achievement and disability groups

Mathematics and reading achievement were assessed using the Numerical Operations and Oral Reading Fluency subtests from the *Wechsler Individual Achievement Test–Third Edition* (Wechsler, 2009), respectively. The Numerical Operations items for students of this age included basic arithmetic and continued through fractions, algebra, geometry and calculus, solved with pencil and paper. For Oral Reading Fluency, the student read two passages (one at a time) under a time limit. Reading errors (added words, misstated words) were recorded by the experimenter and independently verified by review of an audio-recording of the read passages. The scores were reading accuracy [total word count – (total addition errors + total other errors)] and oral reading fluency [(total word count – total other errors)/total completion time]^∗^60, which were highly correlated (*r* = 0.68, *p* < 0.001).

To identify groups with and without learning difficulties, we first examined the distribution of achievement scores for the current sample, focusing on the 25th percentile (Geary et al., 2007; Murphy et al., 2007). For Numerical Operations, students at the 25th percentile of the current sample were at the 18th percentile based on national norms. Students at or below this cutoff were considered to have mathematics difficulties (*N* = 84). Using the same 18th percentile cutoff based on national norms, 95 students were identified as having reading difficulties based on reading accuracy scores. Only 26 students scored at or below this cutoff for reading fluency and 25 of them were included in the reading difficulties group based on reading accuracy. Thus, the reading difficulties group was determined based solely on reading accuracy, although we still conducted individual differences analyses for Oral Reading Fluency scores.

Forty-six students fell into both groups and were classified as having comorbid learning difficulties [National Percentile Ranks = 6.60 (*SD* = 4.70) and 8.18 (*SD* = 5.46) for mathematics and reading, respectively]. Forty-nine students fell into the reading but not mathematics group and were classified as having reading difficulties [National Percentile Ranks = 47.45 (*SD* = 21.91) and 9.06 (*SD* = 5.38) for mathematics and reading, respectively]. Thirty-eight students fell into the mathematics but not reading group and were classified as having mathematics difficulties [National Percentile Ranks = 10.58 (*SD* = 5.37) and 40.53 (*SD* = 17.55) for mathematics and reading, respectively]. As a comparison group, we used the 182 students who did not fall into any of the difficulties groups [National Percentile Ranks = 64.60 (*SD* = 26.31) and 48.35 (*SD* = 18.65) for mathematics and reading, respectively].

#### Cognitive Measures

All of the tasks are standard measures of short-term and working memory, verbal memory, and various aspects of spatial ability. Most of the tasks were administered on iPads using customized programs developed through Inquisit by Millisecond.^2^ The verbal memory task was administered using a customized program developed in Qualtrics;^3^ manuals are available on OSF.^4^ With the exception of N-back which was only administered in seventh grade (due to time constraints), all tasks were administered in seventh and eighth grade. The score was the mean across the two grades, which should provide a more stable estimate of their abilities in these areas than will scores for any single grade. We estimated the reliabilities (ρ) of these summary scores using the Spearman–Brown Prophecy formula applied to the test–retest correlations across grades.

The assessment of verbal short-term memory included a measure of passive memory for strings of words, as well as the more standard forward digit span measure, whereas the assessment of visuospatial memory included a forward spatial span task (Gathercole et al., 2004). The working memory measures involved the active retention of information, while processing other information and included the standard backward digit span measure and N-back. The latter engages brain regions typically associated with working memory but are not identical to those engaged in the digit span task (Yaple and Arsalidou, 2018). The inclusion of both measures thus provided a broader assessment of working memory than the inclusion of only one of them. The broad assessment of working memory is potentially important because it is more consistently related to outcomes in mathematics than are other executive functions (e.g., inhibition; Bull and Lee, 2014). The spatial ability measures assessed visuospatial attention and the ability to generate and manipulate images (Hegarty, 2018), and these too are predictive of outcomes in mathematics (Casey et al., 1997; Kyttälä and Lehto, 2008).

##### Digit span

The students hear a sequence of digits presented at 1 s intervals, starting with three digits for the forward assessment and two digits for the backward assessment. The task is to recall the digit list in order (in either a forward or backward manner, respectively) by tapping the digits on a circle of digits displayed on the iPad screen. If the response is correct, the student moves up to the next level. If the response is incorrect, the same level is presented a second time. If a consecutive error occurs, the student moves down to a lower level. Each direction (forward and then backward) ends after 14 trials. The score was the highest digit span correctly recalled before making two consecutive errors at the same span length. Estimated reliabilities for the current sample were adequate for both forward (ρ = 0.68) and backward (ρ = 0.73) digit span.

##### Verbal memory

The verbal memory measure was taken from a longer proactive inhibition task. The student listens to a recording of a set of four animal words, presented in 1-s intervals using the iPad speakers. To prevent rehearsal, the student immediately names colors from a sheet with rows of different colors for 10 s. After 10 s, a tone prompts the student to recall the words, in order. Responses are recorded by the experimenter using Qualtrics on the iPad. The process is repeated with two new sets of four animal words, and finally with a set of four fruit words. Items were taken from Paivio et al. (1968) and Gilhooly and Logie (1980). The words were chosen based on Imagery (I) and Concreteness (C) ratings (1 to 7 scale), with all scores > 6. The one exception was ‘lime’ (imagery of 5.7), which was included because it was the closest (to 6.0) available one-syllable fruit word. Each quartet included one moderate to high frequency word and three low frequency words (<10/million), and three 1-syllable and one 2-syllable words. All within-list words started with different letters and presentation orders were initially randomized, and subsequently presented in the same order to all students. We used percent correct on the first quartet of words as a measure of short-term verbal memory (ρ = 0.40 for the current sample). The ρ is low but the internal consistency of the measure, based on the eight trials across grades, is adequate (α = 0.66).

We did not use performance on the three other quartets of words because there were (as expected) memory interference effects for these quartets and thus they did not provide measures of basic verbal memory.

##### N-back

Following Jaeggi et al. (2010), students completed an adaptive version of a single N-back task. The student is shown a “target” letter and then a sequence of 20 randomly determined stimulus letters (all consonants; 6 are target; 14 are not) and asked to indicate whether the currently presented letter is a target by tapping a key, or is not a target by not responding. The target letter could be the first stimuli presented (*N* = 0) or could be the same as the one that preceded it (*N* = 1) or the same as one presented in the 2 (*N* = 2) or 3 (*N* = 3) trials that preceded it.

For each trial a letter is presented for 500 ms, followed by a 2,500 ms blank screen, and then by the next letter in the sequence. Students have the entire 3,000 ms to respond by tapping a key if they detect a target. After three 10-item practice blocks for levels *N* = 0 to *N* = 2, all participants start on level *N* = 0. Depending on performance, they move up, stay on the current level, or move down a level for five total blocks (<3 errors – move up; 3–5 errors – repeat level; >5 errors – move down). Performance feedback (percent correct) is displayed after each block. Hits (H), Misses, False Alarms (FA), and Correct Rejections are recorded and summarized by block. The score is (H – FA)/(total blocks). The estimated split-half reliability for the current sample was 0.74.

##### Spatial span

Spatial span was assessed using the forward Corsi Block Tapping Task (Kessels et al., 2000). Students are presented with a display of nine squares that appear to be randomly arranged. The squares “light up” in a pre-determined sequence, and the task is to tap on the squares in the same order they were lit. The sequence length starts at two squares and could increase to up to nine squares. Students have two attempts at each sequence length. If one of the sequences is recalled correctly, the next sequence level begins; if both sequences at the same level are recalled incorrectly, the task is terminated. The score is the total number of correctly recalled sequences across the whole task (ρ = 0.67 for the current sample).

##### Spatial ability

Judgment of Line Angle and Position Test (JLAP) was the first spatial measure (Collaer et al., 2007), and assesses visuospatial attention (Benton et al., 1978). The task requires students to match the angle of the single presented line to 1 of 15-line options in an array at the bottom center of the iPad screen. The 20 test items are presented one at a time, and the student uses the touch screen to select the matching angle. Each stimulus is presented for up to 10 s, and when a selection is made, a reaction time is recorded, and the next stimulus is immediately presented. The outcome is the number correct (ρ = 0.71 for the current sample).

The Mental Rotation Task (MRT-A; Peters et al., 1995) was the second spatial measure, and assesses the ability to generate and manipulate images (Hegarty, 2018). On each trial, the student views images of 3D drawings of 10 connected cubes. For each trial, there is one target and four choice options, and the task is to select the two options that are rotations of the target figure. After four self-paced practice problems, students are presented with 24 problems in two blocks of 12 problems each (3 min per block). The score is the number of problems on which the student chose both correct options (ρ = 0.83 for the current sample).

#### Non-cognitive Measures

Due to assessment delays related to the Covid-19 pandemic, we only have attitudes and anxiety data for seventh grade.

##### Academic attitudes

Mathematics and English attitudes were assessed using measures from the Michigan Study of Adolescent and Adult Transitions.^5^ The measures are designed to assess students’ self-efficacy in and their beliefs about the long-term utility of these areas (Meece et al., 1990; Eccles and Wigfield, 2002). The mathematics measure included seven items on a 1-to-7 Likert scale; e.g., “How much do you like doing math?” rated from 1 (a little) to 7 (a lot), with the six English items being similar.

Previous analyses using an exploratory principle components factor analysis (EFA), as well as parallel and MAP analyses (R Core Team, 2017), indicated that the mathematics items defined two factors and the English items one factor (Geary et al., 2020). For mathematics attitudes, the loadings of individual items on their respective factors were consistent with distinct utility (Items 1 to 4, inclusive) and self-efficacy (Items 5 to 7) dimensions. The scores were the sum of the corresponding items (α = 0.71 for utility, and 0.78 for self-efficacy). The English attitudes score was the mean of the six items (α = 0.83).

##### Mathematics anxiety

The 10 items were adapted from Hopko et al. (2003). Each item (e.g., “Taking an examination in a math course”) was rated on a 1 (low anxiety) to 5 (high anxiety) scale (Geary et al., 2019). All three analyses (i.e., EFA, MAP, parallel) indicated two factors. The first included five items that involved learning mathematics (e.g., “Watching a teacher work an algebraic equation on the board”; items 1, 3, 6, 7, 9) and the second four items that involved some type of evaluation (e.g., “Taking an examination in a math course”; items 2, 4, 5, 8), and the final item (i.e., “In general, how anxious are you about math?”). Composite scores were based on the mean of the five learning anxiety items (α = 0.77) and the five evaluation anxiety items (α = 0.86). The two core factors identified here are consistent with previous findings (Baloglu and Koçak, 2006).

##### In-class attentive behavior

In-class attentive behavior was assessed using the Strength and Weaknesses of ADHD-Symptoms and Normal-Behavior (SWAN) measure (Swanson et al., 2012). The items assess attentional deficits and hyperactivity, but the scores are normally distributed and based on the behavior of a typical student. The nine item (e.g., “Gives close attention to detail and avoids careless mistakes”) attention subscale was distributed to the students’ seventh-grade and eighth-grade mathematics and English language arts teachers who were asked to rate the behavior of the student relative to other students of the same age on a 1 (far below) to 7 (far above) scale. Ratings were consistent across items (αs = 0.98), mathematics and language arts teachers within grades (*r*s = 0.69 to 0.73), and across grades (*r*s = 0.67 to 0.88). Given this consistency, we calculated one in-class attentive behavior score based on mean ratings across teachers and grades (*α* = 0.92).

### Procedure

In seventh grade, the students were administered the intelligence, achievement, attitudes, anxiety, and cognitive measures individually at a quiet location in their school across three 45-min assessments. As shown in Table 1, with the exception of the verbal memory task (due to time constraints), the cognitive measures were administered during the first semester of seventh grade, and the remaining measures during the second semester. In eighth grade, all of the cognitive tasks were assessed in the fall semester and the achievement measures in the spring. In the spring of both grades, students competed the attitudes and anxiety measures and teachers completed the in-class attentive behavior survey.

**TABLE 1:** Age of administration and timing of assessments.

Task name	Seventh grade		Eighth grade	
	Fall	Spring	Fall	Spring
Mean age at test	153	156	164	168
Digit span forward	x		x	
Digit span backward	x		x	
N-back	x			
Spatial span	x		x	
Judgment of Line Angle and Position	x		x	
Mental Rotations Test	x		x	
Verbal memory		x	x	
Intelligence		x		
Oral Reading Fluency				x
Numerical Operations				x
In-class attentive behavior		x		x
Mathematics efficacy		x		x
Mathematics utility		x		x
English attitudes		x		x
Mathematics anxiety for learning		x		x
Mathematics anxiety for evaluation		x		x

*Age is in months, *SD*s range between 4.47 and 4.96 months.*

Parents provided informed written consent, and assent was obtained from adolescents for all assessments. The University of Missouri Institutional Review Board (IRB; Project 2002634, “Algebraic Learning and Cognition”) approved all methods included in this study.

### Analyses

The first goal was to identify common and unique predictors of individual differences in reading and mathematics achievement. To do so, we first used Bayesian regressions to identify the best set of cognitive and non-cognitive predictors of achievement (Gallistel, 2009; Rouder and Morey, 2012). For this we used the *BayesFactor* package in R (v0.9.12-4.2; Morey and Rouder, 2015) with default prior scales for standardized slopes (*r*_*scale*_ = 1/2). Bayes Factors provide information regarding whether the inclusion of specific predictors improves model fit above and beyond other predictors simultaneously considered in the model. This method is more robust than standard linear regression with correlated variables. Bayes Factors are higher when one of two highly correlated variables are included in relation to models containing both or none, providing the ability to compare the relative contribution of individual predictors. In separate analyses, we selected the best combination of cognitive and then non-cognitive predictors of standardized eighth-grade Oral Reading Fluency and Numerical Operations scores. The variables identified from each of these analyses were subsequently used in a follow-up analysis to identify the best combination of cognitive and non-cognitive predictors of these achievement scores. The sequence of analyses provides structured, step-by-step information on the best set of cognitive, non-cognitive, and combined predictors of individual differences in achievement. The results are identical to those that would emerge if all variables were considered simultaneously, but the approach used here provides more information regarding the relative importance of different combinations of cognitive and non-cognitive variables.

The first set of Bayes Factors are noted as MC_m_, where m = the specific set of cognitive (C) predictors in the model (M) and comparisons as BC_mn_, with B representing the comparison ratio of Bayes Factors between models m and n. BC_m__0_ represents a contrast of the selected model to a null model with no predictors. These analyses assess the likelihood of the data for alternative models. For the cognitive measures, the initial analysis included digit span forward, digit span backward, N-back, Corsi, JLAP, MRT, verbal memory, and IQ as potential predictors. The first model identified the most probable subset of these variables as predictors of the achievement outcome. For the non-cognitive measures, we included all of the English and math attitudes and math anxiety variables in the prediction of both math and reading achievement as a way to assess the convergent and discriminant validity of these variables. That is to determine if students are making subject-specific discriminations in their self-reports (they were, below).

For instance, the full model (including all selected cognitive predictors from the first regression) MC_1_ for the prediction of Oral Reading Accuracy included digit span forward, JLAP, verbal memory, and IQ. Each of these predictors were then dropped one-by-one and change in the odds of the model was evaluated. Dropping IQ resulted in model MC_2_ and the comparison to the full model as BC_21_. The latter resulted in a Bayes Factor ratio of 5.17 × 10^6^, meaning the model without IQ was <1% as probable as the model with it. Dropping verbal memory resulted in a model that was 32.35% as probable (MC_31_) or stated differently the model including verbal memory was preferred 3.09 times to 1 over the model without it. Here, lower Bayes Factors indicate greater evidence for a predictor. As a rule of thumb, models that are less than 33% as probable without the variable provide evidence for retaining it, and models that are less than 10% as probable provide strong evidence for retaining it (Jeffreys, 1961; Raftery, 1995). We used the 33% criterion for variable retention, corresponding to a commonly used cutoff for positive evidence (e.g., Bayes factor of three, Kass and Raftery, 1995); stated differently, to be retained the model with the variable had to be preferred at least 3 to 1 over the model without it.

Once the best set of predictors was identified, we used multi-level models to estimate the relative importance of the common and unique predictors of Oral Reading Accuracy and Numerical Operations and Oral Reading Fluency and Numerical Operations scores using Proc Mixed (SAS Institute, 2014). Students were distributed among six schools and there were small but significant school differences for Oral Reading Accuracy, *F*(5,309) = 3.84, *p* = 0.002, *r*^2^ = 0.06, and Numerical Operations, *F*(5,309) = 5.42, *p* < 0.001, *r*^2^ = 0.08. To model these effects, students were assigned as level 1 units and schools as level 2 units in the multi-level models, which allowed intercepts to vary randomly for schools. Achievement scores and predictor variables were centered (*M* = 0, *SD* = 1) and Oral Reading Accuracy (or Fluency) and Numerical Operations scores were nested in an overall achievement variable. Differences across reading and mathematics achievement were estimated with test by predictor interactions. Initially, all variables identified in the Bayesian analyses were included as fixed effects, along with the interactions with test. Non-significant interactions were dropped and changes in model fit were assessed using the Bayesian Information Criterion (smaller values indicate better fit) and negative log likelihood estimate. For nested models, values for the latter can be evaluated using a χ^2^ statistic (Wilks, 1938).

Next, logistic regressions were used to predict inclusion or not in each of the three learning difficulties groups (i.e., comorbid difficulties, reading difficulties, mathematics difficulties) relative to the group of typically achieving students, and inclusion in the mathematics difficulties as compared to the reading difficulties group. The variables used in each regression were based on the results from the Bayesian and multi-level models. These sets of variables provide the best estimate of the combination of factors that predict different forms of learning difficulty and a means to estimate the relative importance of each individual predictor. Moreover, the Cohen’s *d* of the log odds of group membership is identical to the multivariate Mahalanobis distance (i.e., multivariate *d*) and thus provides a multivariate estimate of the magnitude of the differences across the students in the learning difficulties groups and students in the typically achieving group.

## Results

**FIGURE 1:**
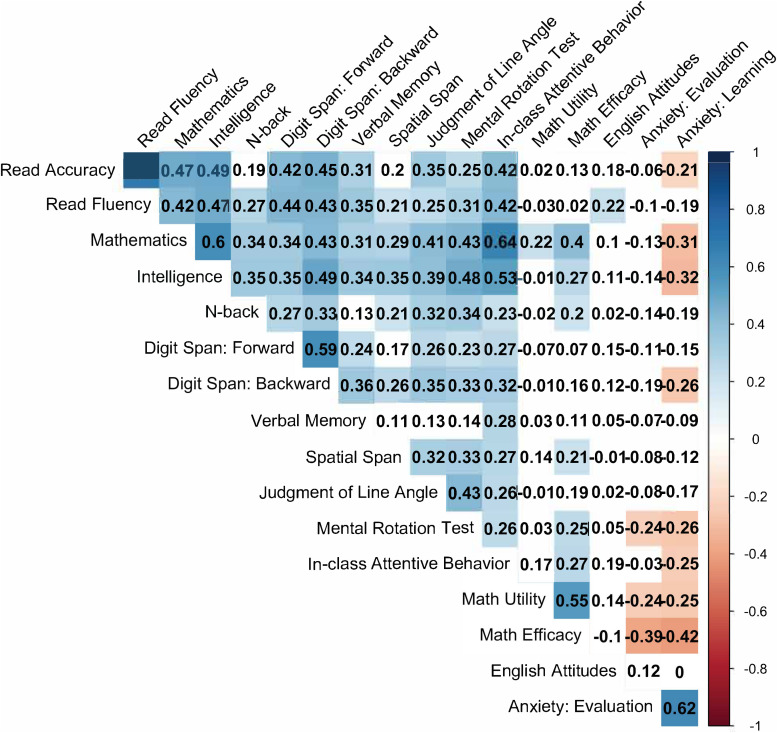
Correlations among predictors and reading and mathematics achievement.

Mean scores across measures are shown in Table 2 for the entire sample and the samples of typically achieving and learning difficulty groups. Whole-sample correlations among the measures are shown in Figure 1.

**TABLE 2:** Means for cognitive and non-cognitive measures.

	Overall (*N* = 315)	Typically achieving (*N* = 182)	Comorbid difficulty (*N* = 46)	*d*	Reading difficulty (*N* = 49)	*d*	Math difficulty (*N* = 38)	*d*
Measure	*M* (*SD*)	*M* (*SD*)	*M* (*SD*)		*M* (*SD*)		*M* (*SD*)	
**Achievement**								
Oral Reading Accuracy	92.71 (12.52)	99.65 (8.49)	77.35 (6.73)	1.78	78.47 (5.96)	1.69	96.42 (7.91)	0.26
Oral Reading Fluency	103.58 (11.79)	108.59 (8.49)	89.93 (10.84)	1.58	96.08 (10.04)	1.06	105.76 (10.87)	0.24
Numerical Operations	98.85 (18.22)	108.64 (14.81)	75.20 (7.16)	1.84	99.37 (9.44)	0.51	79.95 (5.77)	1.57
**Cognitive**								
Intelligence	105.07 (13.09)	110.77 (10.70)	90.33 (10.43)	1.56	102.27 (11.28)	0.65	99.21 (11.01)	0.88
N-back	3.80 (0.76)	3.95 (0.76)	3.38 (0.71)	0.75	3.72 (0.72)	0.30	3.68 (0.66)	0.36
Digit span forward	5.86 (0.99)	6.16 (0.97)	5.14 (0.76)	1.03	5.46 (0.82)	0.71	5.83 (0.90)	0.33
Digit span backward	4.72 (1.12)	5.14 (1.07)	3.78 (0.80)	1.21	4.30 (0.78)	0.75	4.38 (1.06)	0.68
Verbal memory	0.68 (0.23)	0.74 (0.20)	0.53 (0.25)	0.91	0.61 (0.21)	0.57	0.65 (0.24)	0.39
Spatial span	8.83 (1.96)	9.26 (1.91)	7.61 (1.94)	0.84	8.76 (1.65)	0.26	8.34 (1.91)	0.47
Judgment of Line Angle	13.57 (2.86)	14.45 (2.70)	11.74 (2.48)	0.95	12.74 (2.95)	0.59	12.63 (2.32)	0.64
Mental Rotation Test	9.88 (4.31)	11.00 (4.27)	6.41 (2.60)	1.06	9.85 (3.95)	0.27	8.76 (4.17)	0.52
**Non-cognitive**								
In-class attentive behavior	4.90 (1.35)	5.49 (1.07)	3.32 (1.15)	1.61	4.89 (1.03)	0.44	3.97 (1.12)	1.13
Math utility	5.25 (0.97)	5.36 (0.96)	4.89 (0.93)	0.48	5.48 (0.82)	−0.12	4.89 (1.09)	0.48
Math efficacy	5.02 (1.02)	5.23 (0.87)	4.38 (1.01)	0.83	5.18 (0.94)	0.05	4.48 (1.27)	0.74
English attitudes	5.06 (1.11)	5.19 (1.00)	4.88 (1.22)	0.28	4.85 (1.10)	0.31	4.92 (1.38)	0.24
Math anxiety for evaluation	2.61 (0.96)	2.54 (0.95)	2.80 (0.96)	−0.27	2.55 (0.94)	−0.01	2.81 (1.02)	−0.28
Math anxiety for learning	1.71 (0.65)	1.58 (0.55)	2.07 (0.73)	−0.75	1.70 (0.69)	−0.18	1.91 (0.76)	−0.51

**d* = (M_*Typical*_ – M_*Difficulty*_)/pooled *SD*. The achievement and intelligence scores are standardized based on national norms (*M* = 100, *SD* = 15). Raw scores are presented for all other variables.*

### Bayesian Regressions

#### Oral Reading Accuracy

As noted, the best set of cognitive predictors of Oral Reading Accuracy scores were digit span forward, JLAP, verbal memory, and IQ (see Table 3). The BC_m__0_ is very large for this first model and all alternative models, providing strong evidence for some combination of cognitive predictors of Oral Reading Accuracy relative to the null. Dropping IQ and digit span forward resulted in models that were <1% as probable as the models without them. Dropping verbal memory and JLAP resulted in models that were 32.35% (MC_31_) or 14.05% (MC_41_) as probable as the models with them; or stated otherwise, the models including verbal memory and JLAP were preferred 3.09 and 7.12 times to 1 relative to models without them. On the basis of these results, all four variables were retained for the combined analyses.

**TABLE 3:** Bayes factor analyses of predictors of oral reading achievement.

Oral Reading Accuracy			
**Model: Top Cognitive Predictors**	**BC_m__0_**	**Excluded**	**BC_m__1_**

MC_1_ DSF + JLAP + Verbal memory + IQ	6.92 × 10^23^	–	1
MC_2_ DSF + JLAP + Verbal memory	3.58 × 10^18^	IQ	0.0000
MC_3_ DSF + JLAP + IQ	2.24 × 10^23^	Verbal memory	0.3235
MC_4_ DSF + Verbal memory + IQ	9.72 × 10^22^	JLAP	0.1405
MC_5_ + JLAP + Verbal memory + IQ	4.76 × 10^19^	DSF	0.0000

**Model: Top Non-cognitive Predictors**	**BNC_m__0_**	**Excluded**	**BNC_m__1_**

MNC_1_ English attitudes + MAnxLearn + Attentive behavior	1.01 × 10^12^	–	1
MNC_2_ English attitudes + MAnxLearn	2.49 × 10^3^	Attentive behavior	0.0000
MNC_3_ English attitudes + Attentive behavior	5.98 × 10^11^	MAnxLearn	0.5946
MNC_4_ MAnxLearn + Attentive behavior	8.38 × 10^11^	English attitudes	0.8327

**Model: Top Combined Predictors**	**BA_m__0_**	**Excluded**	**BA_m__1_**

MA_1_ DSF + JLAP + Verbal memory + IQ + Attentive behavior	1.37 × 10^25^	–	1
MA_2_ DSF + JLAP + Verbal memory + IQ	6.92 × 10^23^	Attentive behavior	0.0504
MA_3_ DSF + JLAP + Verbal memory + Attentive behavior	1.03 × 10^23^	IQ	0.0075
MA_4_ DSF + JLAP + IQ + Attentive behavior	9.07 × 10^24^	Verbal memory	0.6606
MA_5_ DSF + Verbal memory + IQ + Attentive behavior	2.67 × 10^24^	JLAP	0.1948
MA_6_ JLAP + Verbal memory + IQ + Attentive behavior	2.07 × 10^21^	DSF	0.0002

**Oral Reading Fluency**			

**Model: Top Cognitive Predictors**	**BC_m__0_**	**Excluded**	**BC_m__1_**

MC_1_ DSF + Verbal memory + IQ	2.66 × 10^24^	–	1
MC_2_ DSF + Verbal memory	7.03 × 10^17^	IQ	0.0000
MC_3_ DSF + IQ	4.57 × 10^22^	Verbal memory	0.0172
MC_4_ Verbal memory + IQ	4.45 × 10^18^	DSF	0.0000

**Model: Top Non-cognitive Predictors**	**BC_m__0_**	**Excluded**	**BC_m__1_**

MNC_1_ MUtility + English attitudes + MAnxEval + Attentive behavior	1.22 × 10^14^	–	1
MNC_2_ MUtility + English attitudes + MAnxEval	4.10 × 10^2^	Attentive behavior	0.0000
MNC_3_ MUtility + English attitudes + Attentive behavior	1.56 × 10^13^	MAnxEval	0.1276
MNC_4_ MUtility + MAnxEval + Attentive behavior	1.86 × 10^12^	English attitudes	0.0152
MNC_5_ English attitudes + MAnxEval + Attentive behavior	9.21 × 10^12^	MUtility	0.0752

**Model: Top Combined Predictors**	**BA_m__0_**	**Excluded**	**BA_m__1_**

MA_1_ DSF + Verbal memory + IQ + English attitudes + Attentive behavior	3.30 × 10^26^	–	1
MA_2_ DSF + Verbal memory + IQ + English attitudes	2.40 × 10^25^	Attentive behavior	0.0727
MA_3_ DSF + Verbal memory + IQ + Attentive behavior	1.06 × 10^26^	English Attitudes	0.3217
MA_4_ DSF + Verbal memory + English attitudes + Attentive behavior	8.01 × 10^23^	IQ	0.0024
MA_5_ DSF + IQ + English attitudes + Attentive behavior	2.21 × 10^25^	Verbal memory	0.0366
MA_6_ Verbal memory + IQ + English attitudes + Attentive behavior	5.42 × 10^21^	DSF	0.0000

*DSF, Digit Span Forward; JLAP, Judgment of Line Angle and Position Test; MRT, Mental Rotation Test; MAnxLearn, Mathematics Anxiety for Learning; MAnxEval, Mathematics Anxiety for Evaluation; MUtility, Mathematics Utility; MC, Models for cognitive variables; MNC, Models for non-cognitive variables; MA, Models for all, that is, top cognitive and non-cognitive variables.*

The second section of Table 3 indicates that the best set of non-cognitive predictors of Oral Reading Accuracy included mathematics anxiety for learning, English attitudes, and in-class attentive behavior. Dropping the latter resulted in a model (MNC_2_) that was <1% as probable as the model with it. However, dropping mathematics anxiety and English attitudes resulted in models that were 59.46% (MNC_3_) and 83.27% (MNC_4_), respectively, as probable as the models with them respectively; or stated otherwise, the models including mathematics anxiety and English attitudes were preferred 1.68 and 1.20 times to 1 relative to models without them. The two latter results indicate that inclusion of these variables does not add substantively to the prediction of Oral Reading Accuracy and thus only in-class attentive behavior was retained for the combined analyses.

The combined analysis included digit span forward, JLAP, verbal memory, IQ, and in-class attentive behavior. As shown in Table 3, the best model included all five predictors. However, dropping verbal memory resulted a model that was 66.06% (MA_4_) as probable as the model with it, and thus this variable was dropped. The other models provide evidence for the retention of the remaining variables.

#### Oral Reading Fluency

The bottom sections of Table 3 show the Bayesian results for the prediction of Oral Reading Fluency scores. The best set of cognitive predictors included digit span forward, verbal memory, and IQ. The BC_m__0_ is very large for this first model and all alternative models, providing strong evidence for some combination of cognitive predictors of Oral Reading Fluency. As can be seen, there is strong evidence for the retention of each of these variables.

The next section of Table 3 shows that the top set of non-cognitive predictors of Oral Reading Fluency included mathematics utility, English Attitudes, mathematics anxiety for evaluation, and in-class attentive behavior. Dropping each of these variables in turn resulted in models that were <12.76% as probable as the model with them, or models with each of these predictors are preferred at least 7.84 to 1 over models without them. Thus, all were retained for the combined analysis.

The combined analyses included digit span forward, verbal memory, IQ, mathematics utility, English Attitudes, mathematics anxiety for evaluation, and in-class attentive behavior. As shown in the final section of Table 3, all of these predictors were in the best model, except for mathematics utility and mathematics anxiety for evaluation. Dropping each of the remaining predictors in turn resulted in models that were <32.17% as probable as the models with them; or stated otherwise, the models with each of these predictors were preferred at least 3.11 to 1 over the models without them.

In all, digit span forward, IQ, and in-class attentive behavior were common predictors of Oral Reading Accuracy and Oral Reading Fluency, whereas JLAP was unique to the former and Verbal Memory and English Attitudes to the latter.

#### Numerical Operations

A summary of the Bayesian models for the prediction of Numerical Operations scores is presented in Table 4. The first of these sections shows that the best set of cognitive predictors included digit span forward, JLAP, MRT, verbal memory, and IQ. Dropping IQ resulted in a substantive reduction in model fit (<1% as probable as the model with it). Dropping verbal memory (55.09% as probable, or the model with it is preferred 1.82 to 1 relative to the model without it) and digit span forward (79.40% as probable, or the model with it is preferred 1.26 to 1 relative to the model without it) resulted in models that were not substantively different than the models with them. There was positive evidence for the retention of MRT (19.09% as probable, or the model with it is preferred 5.24 to 1 to the model without it) and JLAP (10.31% as probable, or the model with it is preferred 9.7 to 1 to the model without it). On the basis of these findings, JLAP, MRT, and IQ were retained for the final analyses.

**TABLE 4:** Bayes factor analyses of predictors of mathematics achievement.

Numerical Operations			
**Model: Top Cognitive Predictors**	**BC_m__0_**	**Excluded**	**BC_m__1_**

MC_1_ DSF + JLAP + MRT + Verbal memory + IQ	1.80 × 10^32^	–	1
MC_2_ DSF + JLAP + MRT + Verbal memory	3.08 × 10^22^	IQ	0.0000
MC_3_ DSF + JLAP + MRT + IQ	9.94 × 10^31^	Verbal memory	0.5509
MC_4_ DSF + JLAP + Verbal memory + IQ	3.45 × 10^31^	MRT	0.1909
MC_5_ DSF + MRT + Verbal memory + IQ	1.86 × 10^31^	JLAP	0.1031
MC_6_ JLAP + MRT + Verbal memory + IQ	1.43 × 10^32^	DSF	0.7940

**Model: Top Non-cognitive Predictors**	**BC_m__0_**	**Excluded**	**BC_m__1_**

MNC_1_ Math efficacy + Attentive behavior	3.23 × 10^40^	–	1
MNC_2_ Math efficacy	7.32 × 10^10^	Attentive behavior	0.0000
MNC_3_ Attentive behavior	4.17 × 10^34^	Math efficacy	0.0000

**Model: Top Combined Predictors**	**BA_m__0_**	**Excluded**	**BA_m__1_**

MA_1_ JLAP + MRT + IQ + Math Efficacy + Attentive behavior	7.81 × 10^51^	–	1
MA_2_ JLAP + MRT + IQ + Math efficacy	3.12 × 10^36^	Attentive behavior	0.0000
MA_3_ JLAP + MRT + IQ + Attentive behavior	3.54 × 10^48^	Math efficacy	0.0005
MA_4_ JLAP + MRT + Math efficacy + Attentive behavior	3.72 × 10^48^	IQ	0.0005
MA_5_ JLAP + IQ + Math efficacy + Attentive behavior	2.07 × 10^51^	MRT	0.2657
MA_6_ MRT + IQ + Math efficacy + Attentive behavior	1.16 × 10^51^	JLAP	0.1485

*DSF, Digit Span Forward; JLAP, Judgment of Line Angle and Position Test; MRT, Mental Rotation Test; MC, Models for cognitive variables; MNC, Models for non-cognitive variables; MA, Models for all, that is, top cognitive and non-cognitive variables.*

As shown in Table 4, there was strong evidence for the inclusion of mathematics self-efficacy and in-class attentive behavior among the non-cognitive predictors of Numerical Operations scores. Thus, the final combined analyses included JLAP, MRT, IQ, mathematics self-efficacy, and in-class attentive behavior. The best model included all of these variables. Dropping each of the predictors resulted in models that were less than 26.57% as probable as models with them; in other words, the models with them were preferred at least 3.76 to 1 over the models without them.

#### Intelligence and Working Memory

On the basis of prior research, we anticipated one or several of the working memory measures would emerge as predictors of reading and mathematics achievement (Swanson et al., 2009b; Lee and Bull, 2016; Geary et al., 2017). This was the case for Oral Reading Fluency but not for Oral Reading Accuracy or Numerical Operations scores. One possibility is that inclusion of IQ in the analyses obscured any relation between working memory and these outcomes, given the correlation between performance on IQ and working memory measures (Ackerman et al., 2005). To assess this possibility, we conducted *post hoc* analyses for Oral Reading Accuracy and Numerical Operations, dropping IQ.

For Oral Reading Accuracy, dropping IQ resulted in the identification of digit span backward as a predictor, along with digit span forward, JLAP and in-class attentive behavior as in the original analyses. Similarly, there was no change in the best model for predicting Numerical Operations scores, except that digit span backward replaced IQ.

### Multi-Level Models

#### Oral Reading Accuracy and Numerical Operations

The Bayesian analyses identified IQ, in-class attentive behavior, and JLAP as common predictors of Oral Reading Accuracy and Numerical Operations scores, and digit span forward as unique to reading achievement and MRT and mathematics self-efficacy as unique to mathematics achievement. These six variables along with achievement test (reading = 0, mathematics = 1) and test by variable interactions were included in the multi-level models.

The full model revealed non-significant interactions between test and IQ (*p* = 0.391) and test and JLAP (*p* = 0.599). Dropping these two interactions did not substantively change overall model fit [ΔBIC = 2.4, χ^2^(2) = 1.1, *p* = 0.577]. The estimates associated with the model that did not include these interactions are shown in Table 5. The highly significant main effects, without significant interactions for IQ and JLAP (*p*s < 0.001), confirm the importance of these variables in the prediction of overall achievement, that is, achievement across reading and mathematics.

**TABLE 5:** Estimates from multi-level model for Oral Reading Accuracy and Numerical Operations.

Effect	Estimate (*se*)	*t*-Test	*p*
**Fixed effects**			
Intercept	−0.012 (0.06)	−0.18	0.867
Intelligence	0.234 (0.04)	5.71	0.000
In-class attentive behavior	0.390 (0.05)	8.26	0.000
JLAP	0.134 (0.04)	3.81	0.000
Digit span forward	0.078 (0.04)	1.78	0.077
Mental Rotation Test (MRT)	0.100 (0.05)	2.14	0.033
Mathematics efficacy	0.171 (0.04)	3.93	0.000
Test by in-class attentive behavior	−0.184 (0.06)	−3.13	0.002
Test by digit span forward	0.176 (0.06)	3.09	0.002
Test by MRT	−0.121 (0.06)	2.09	0.037
Test by mathematics efficacy	−0.205 (0.06)	−3.58	0.000
**Random effects**			
Intercepts: Schools	0.015 (0.01)	1.11	0.133
Intercepts: Students in schools	0.061 (0.03)	2.07	0.019
Residual	0.459 (0.04)	12.55	0.000

*JLAP, Judgment of Line Angle and Position.*

The significant interactions indicate that the relative importance of the predictor varies across reading and mathematics achievement, with positive estimates indicating larger effects in the prediction of reading achievement and negative estimates indicating larger effects in the prediction of mathematics achievement. The interactions are consistent with the Bayesian analyses, with digit span forward being relatively more important for the prediction of reading accuracy and MRT and mathematics self-efficacy for mathematics achievement. In-class attentive behavior predicts reading and mathematics achievement, but the interaction reveals that it is relatively more important for mathematics.

#### Oral Reading Fluency and Numerical Operations

The Bayesian analyses identified IQ and in-class attentive behavior as common predictors of Oral Reading Fluency and Numerical Operations scores. Digit span forward, verbal memory, and English Attitudes were unique to reading fluency, whereas JLAP, MRT and mathematics self-efficacy were unique to mathematics achievement. These eight variables along with achievement test (reading = 0, mathematics = 1) and test by variable interactions were included in the multi-level models.

The full model revealed non-significant interactions between test and IQ (*p* = 0.877) and test and MRT (*p* = 0.953). Dropping these two interactions did not substantively change overall model fit [ΔBIC = 1.8, χ^2^(2) < 1, *p* < 0.001]. The estimates associated with the model that did not include these interactions are shown in Table 6. The highly significant main effects, without significant interactions for IQ and MRT (*p*s < 0.001), indicate that these variables predicted achievement in both domains.

**TABLE 6:** Estimates from multi-level model for Oral Reading Fluency and Numerical Operations.

Effect	Estimate (*se*)	*t*-Test	*p*
**Fixed effects**			
Intercept	−0.007 (0.05)	−0.14	0.893
Intelligence	0.193 (0.04)	4.82	0.000
In-class attentive behavior	0.391 (0.05)	8.21	0.000
Digit span forward	0.077 (0.04)	1.76	0.079
Verbal memory	0.059 (0.04)	1.38	0.167
English attitudes	0.006 (0.04)	0.15	0.881
JLAP	0.116 (0.04)	2.61	0.010
Mental Rotation Test (MRT)	0.119 (0.04)	3.38	0.001
Mathematics efficacy	0.179 (0.04)	4.18	0.000
Test by in-class attentive behavior	−0.198 (0.06)	−3.20	0.002
Test by digit span forward	0.164 (0.06)	2.77	0.006
Test by verbal memory	0.107 (0.06)	1.84	0.067
Test by English attitudes	0.099 (0.06)	1.75	0.080
Test by JLAP	−0.106 (0.06)	−1.82	0.070
Test by mathematics efficacy	−0.321 (0.06)	−5.55	0.000
**Random effects**			
Intercepts: Schools	0.007 (0.01)	0.89	0.186
Intercepts: Students in schools	0.033 (0.03)	1.17	0.121
Residual	0.468 (0.04)	12.55	0.000

*JLAP, Judgment of Line Angle and Position.*

Again, the significant interactions indicate that the relative importance of the predictor varies across reading and mathematics achievement, with positive estimates indicating larger effects in the prediction of reading fluency and negative estimates indicating larger effects in the prediction of mathematics achievement. The interactions indicate stronger relations between in-class attentive behavior, JLAP, and mathematics efficacy and mathematics achievement than reading fluency. In contrast, digit span forward, verbal memory, and English attitudes were more strongly related to reading fluency than to mathematics achievement.

### Logistic Regressions

#### Comorbid Learning Difficulties

The first logistic regression included the common predictors of reading and mathematics achievement, that is, IQ, in-class attentive behavior, and JLAP. The overall model was highly significant, Wald χ^2^(3) = 48.01, *p* < 0.001, and correctly classified 95.5% of the students as having comorbid learning difficulties or not. However, the estimate for JLAP was not significant (*p* = 0.070) and thus the regression was rerun with only IQ and in-class attentive behavior.

The resulting model was highly significant, Wald χ^2^(2) = 48.32, *p* < 0.001, as were the effects for IQ and in-class attentive behavior (*p*s < 0.001). One *SD* increases in IQ and in-class attentive behavior resulted in 4.6-fold [95% confidence interval (CI) = 2.4, 8.7] and 4.7-fold [CI = 2.5, 9.0] increases in the odds of being in the typically achieving group, respectively. The combination correctly classified 94.6% of students as having comorbid learning difficulties or not, which is equivalent to a very large multivariate effect, *D* = 2.44 [CI = 2.03, 2.85]. As a comparison, the univariate effect sizes for IQ (*d* = 1.56) and in-class attentive behavior (*d* = 1.61) were large, as shown in Table 2, but smaller than the combined effect.

#### Reading Difficulties

The first logistic regression included the best predictors of reading achievement identified in the prior analyses, that is, IQ, in-class attentive behavior, JLAP, and digit span forward. The overall model was highly significant, Wald χ^2^(4) = 32.52, *p* < 0.001, and correctly classified 79% of the students as having reading difficulties or not. However, the estimate for in-class attentive behavior was not significant (*p* = 0.077) and thus the regression was rerun with only IQ, JLAP, and digit span forward.

The resulting model was highly significant, Wald χ^2^(3) = 30.78, *p* < 0.001, as were the individual effects (*p*s < 0.05). One *SD* increases in IQ, JLAP, and digit span forward resulted in 1.9-fold [CI = 1.2, 2.9], 1.5-fold [CI = 1.0, 2.2], and 2-fold [CI = 1.3, 3.0] increases in the odds of being in the typically achieving group, respectively. The combination correctly classified 78% of students as having reading difficulties or not, which is equivalent to a large multivariate effect *D* = 1.08 [CI = 0.74, 1.41]. As a comparison, the univariate effect sizes for IQ (*d* = 0.65), JLAP (*d* = 0.59), and digit span forward (*d* = 0.71) were moderate and smaller than the combined effect.

#### Mathematics Difficulties

The first logistic regression included the best predictors of mathematics achievement identified in the prior analyses, that is, IQ, in-class attentive behavior, JLAP, MRT, and mathematics self-efficacy. The overall model was highly significant, Wald χ^2^(5) = 39.80, *p* < 0.001, and correctly classified 87.7% of the students as having mathematics difficulties or not. However, the estimates for JLAP (*p* = 0.107) and MRT (*p* = 0.772) were not significant.

Dropping MRT resulted in a significant effect for JLAP (*p* = 0.044), but a substantive decrease in the percentage (79%) of students who were correctly classified. Dropping other individual variables indicated that the most parsimonious model only included IQ and in-class attentive behavior, Wald χ^2^(2) = 38.57, *p* < 0.001. One *SD* increases in IQ and in-class attentive behavior resulted in 2.2-fold [CI = 1.4, 3.7] and 3.6-fold [CI = 2.1, 6.2] increases in the odds of being in the typically achieving group. The combination correctly classified 86.8% of students as having mathematics difficulties or not, which is equivalent to a large multivariate effect, *D* = 1.59 [CI = 1.20, 1.97]. As a comparison, the univariate effect sizes for IQ (*d* = 0.88) and in-class attentive behavior (*d* = 1.13) were large, but smaller than the combined effect.

#### Mathematics Versus Reading Difficulties

The first logistic regression included the best predictors of mathematics or reading achievement identified in prior analyses, that is, IQ, in-class attentive behavior, forward digit span, JLAP, MRT, and mathematics self-efficacy. The overall model was significant, Wald χ^2^(6) = 17.58, *p* = 0.007, and correctly classified 79.1% of the students as having mathematics rather than reading difficulties. However, the estimates for IQ (*p* = 0.794), JLAP (*p* = 0.657) and MRT (*p* = 0.566) were not significant and thus dropped.

The follow-up regression was significant, Wald χ^2^(3) = 17.19, *p* < 0.001. The individual estimates for in-class attentive behavior (*p* < 0.001) and mathematics self-efficacy (*p* = 0.041) were significant and the estimate for forward digit span was a trend (*p* = 0.065). One *SD* increases in in-class attentive behavior and mathematics self-efficacy resulted in 2.7-fold [CI = 1.5, 4.8] and 1.7-fold [CI = 1.02, 2.93) decreases, respectively, in the odds of being in the mathematics difficulties group. A 1 *SD* increase in forward digit span, in contrast, resulted in a 1.6-fold [CI = 0.97, 2.7] decrease in the odds of being in the reading difficulties group. The combination correctly classified 78.5% of students as having mathematics or reading difficulties, which is equivalent to a large multivariate effect, *D* = 1.16 [CI = 0.66, 1.65].

### In-Class Attentive Behavior

The above analyses indicated that in-class attentive behavior is an important predictor of individual differences in academic achievement and contributes to comorbid and mathematics learning difficulties. In a *post hoc* analyses, we used Bayesian regressions to identify the best set of cognitive and non-cognitive predictors of in-class attentive behavior, which allowed for inferences about the factors that might contribute to students’ disengagement in classroom learning.

As shown in Table 6, the top cognitive model included verbal memory and IQ, but dropping the former resulted in little change in model fit. The model without verbal memory was 86.09% as probable as the one with it, or the model with it was preferred only 1.16 to 1 over the model without it. The top non-cognitive predictors included mathematics self-efficacy, English attitudes, mathematics anxiety for evaluation, and mathematics anxiety for learning. Dropping each of these variables in turn resulted in models that were less than 5% as probable as the models with them, and thus all of them were kept for the combined analyses.

The combined analysis included mathematics self-efficacy, English attitudes, mathematics anxiety for evaluation, mathematics anxiety for learning, and IQ, and the best model included all of them. However, as shown in Table 7, the inclusion of mathematics anxiety for learning did not add substantively to the prediction of in-class attentive behavior and thus was dropped.

**TABLE 7:** Bayes factor analyses of predictors of in-class attentive behavior.

Model: Top Cognitive Predictors	BC_m__0_	Excluded	BC_m__1_
MC_1_ Verbal memory + IQ	6.91 × 10^20^	–	1
MC_2_ Verbal memory	3.13 × 10^4^	IQ	0.0000
MC_3_ IQ	5.95 × 10^20^	Verbal memory	0.8609

**Model: Top Non-cognitive Predictors**	**BNC_m__0_**	**Excluded**	**BNC_m__1_**

MNC_1_ Math efficacy + EngAtt + MAnxEval + MAnxLearn	3.10 × 10^8^	–	1
MNC_2_ Math efficacy + EngAtt + MAnxEval	9.40 × 10^5^	MAnxLearn	0.0030
MNC_3_ Math efficacy + EngAtt + MAnxLearn	1.35 × 10^7^	MAnxEval	0.0436
MNC_4_ Math efficacy + MAnxEval + MAnxLearn	4.54 × 10^6^	EngAtt	0.0146
MNC_5_ EngAtt + MAnxEval + MAnxLearn	2.18 × 10^5^	Math efficacy	0.0007

**Model: Top Combined Predictors**	**BA_m__0_**	**Excluded**	**BA_m__1_**

MA_1_ Math efficacy + EngAtt + MAnxEval + MAnxLearn + IQ	3.99 × 10^22^	–	1
MA_2_ Math efficacy + EngAtt + MAnxEval + MAnxLearn	3.10 × 10^8^	IQ	0.0000
MA_3_ Math efficacy + EngAtt + MAnxEval + + IQ	2.58 × 10^22^	MAnxLearn	0.6464
MA_4_ Math efficacy + EngAtt + + MAnxLearn + IQ	6.80 × 10^21^	MAnxEval	0.1705
MA_5_ Math efficacy + + MAnxEval + MAnxLearn + IQ	5.13 × 10^21^	EngAtt	0.1286
MA_6_ + EngAtt + MAnxEval + MAnxLearn + IQ	2.22 × 10^21^	Math efficacy	0.0556

*MAnxLearn, Mathematics Anxiety for Learning; EngAtt, English Attitudes; MAnxEval, Mathematics Anxiety for Evaluation. MC, Models for cognitive variables; MNC, Models for non-cognitive variables; MA, Models for all, that is, top cognitive and non-cognitive variables.*

A follow-up regression revealed that these four variables explained 33% of the variance in in-class attentive behavior, *F*(4,310) = 37.35, *p* < 0.001. The largest effect was for IQ, β = 0.47, *t*_(__310__)_ = 9.68, *p* < 0.001, followed by mathematics self-efficacy, β = 0.19, *t*_(__310__)_ = 3.62, *p* < 0.001, English attitudes, β = 0.14, *t*_(__310__)_ = 3.03, *p* = 0.003, and mathematics anxiety for evaluation, β = 0.09, *t*_(__310__)_ = 1.84, *p* = 0.068. IQ alone explained 28% of the variance, *F*(1,313) = 120.93, *p* < 0.001, whereas the combination of the three non-cognitive variables (without IQ) explained 12% of the variance in in-class attentive behavior, *F*(3,311) = 14.29, *p* < 0.001.

Overall, students with higher intelligence and mathematics self-efficacy, along with more positive attitudes toward English or language arts and more concern about their performance on mathematics evaluations were more attentive in classrooms.

## Discussion

The current study provided a comprehensive analysis of the common and unique predictors of individual differences in reading and mathematics achievement and learning difficulties in these domains. The results indicate that there are common domain-general cognitive abilities and non-cognitive factors that contribute to individual and group differences in reading and mathematics achievement, as well as factors that are unique to each of them. We discuss the details and implications of these results in terms of individual differences in achievement and with respect to students with learning difficulties.

### Individual Differences in Achievement

The finding that intelligence emerged as a common predictor of reading accuracy, reading fluency, and mathematics achievement is not surprising, given previous findings (Deary et al., 2007; Peng et al., 2019). As noted, on the basis of these findings we anticipated that one or several of the commonly used working memory measures (e.g., backward digit span) would emerge as predictors of both reading and mathematics achievement (Swanson et al., 2009a; Lee and Bull, 2016; Geary et al., 2017), but this was not the case. One potential reason is the well-documented correlation between performance on intelligence and working memory measures (Ackerman et al., 2005). Indeed, dropping IQ resulted in the emergence of working memory (i.e., digit span backward) as a predictor of both reading accuracy and mathematics achievement. In other words, working memory contributes to variation in reading and mathematics achievement, as found in many previous studies, but the associated variance is captured by intelligence. The results confirm that the combination of strong working memory abilities and intelligence indexes the ease of learning academic material (Cattell, 1963; Geary, 2005, 2008).

In keeping with prior studies, in-class attentive behavior also emerged as an important predictor of achievement but more so for mathematics than for reading accuracy or reading fluency (Geary et al., 2013; Fuchs et al., 2016). The pattern likely follows from the importance of attending to classroom lectures for learning mathematical content. In other words, the mathematics achievement measure assessed knowledge that is imparted, at least in part, in the context of classroom instruction and inattention in the classroom is related to slower learning of mathematics (Stigler et al., 1987). The reading achievement measure, in contrast, assessed oral reading that is likely not as dependent on day-to-day attention in classroom settings. Even so, teacher-rated in-class attentive behavior was predictive of individual differences in oral reading accuracy. If the in-attentive behavior reported by teachers is expressed during oral reading, then we would expect less fluent reading and more reading errors, as we found. Interventions that focus students’ attention on each grapheme in words as they read are helpful for reducing such errors (McCandliss et al., 2003).

We also anticipated that one or several of the spatial measures would emerge as stronger predictors of mathematics than reading achievement, and the Bayesian analyses showed that this was the case for the Mental Rotation Test (Casey et al., 1997; Kyttälä and Lehto, 2008). Among other things, the MRT assesses ease of generating mental images (Hegarty, 2018) that in turn could facilitate comprehension of certain types of mathematics (e.g., slopes, number line, parallel lines) and might also contribute to the ability to use spatial strategies during mathematical problem solving (Johnson, 1984). Any such relations would be more important for some types of mathematics than others (Kyttälä and Lehto, 2008), but our mathematics achievement measure does not allow for this type of fine-grain assessment.

Performance on the Judgment of Line Angle and Position Test (Collaer et al., 2007), a measure of visuospatial attention (Tranel et al., 2009), also emerged as a predictor of individual differences in mathematics achievement. Consistent with this finding, prior studies indicate that the visuospatial abilities assessed by this measure are important for discriminating the relative magnitudes of numerals and for positioning them on the number line (Longo and Lourenco, 2007; Zorzi et al., 2012).

However, performance on the JLAP was just as important in predicting oral reading accuracy as mathematics achievement, indicating that visuospatial attention is not uniquely related to mathematics learning. Indeed, prior studies have found that deficits in visuospatial attention contribute to reading difficulties, including word reading errors (Friedrich et al., 1985; Valdois et al., 2019). Valdois et al.’s (2019) study suggests that the deficits are associated with the top-down control of visual attention. Deficits in control of visual attention would hamper the processing of visually and sequentially presented details, which would make accurate reading and many aspects of mathematics (e.g., processing an equation) error prone. At the same time, oral reading fluency was not related to JLAP performance, indicating the students who committed reading errors were still able to appear to fluently read, although they often generated words that were not actually in the text.

In addition to the Mental Rotation Test, mathematics self-efficacy was more important in the prediction of mathematics than reading achievement, and English attitudes were more strongly related to reading fluency than to mathematics achievement or reading accuracy The pattern indicates that students were differentiating between their competencies in mathematics and reading, and that perceived effort during the act of reading (i.e., fluency) might be more important in shaping associated attitudes than reading accuracy. The cause-effect relation between attitudes and achievement cannot, however, be determined from these results. On the basis of prior results, it is likely that the relation emerged because students are aware of their relative performance in mathematics and reading and this in turn influenced their attitudes in these areas (Talsma et al., 2018; Geary et al., 2019).

Verbal short-term memory, as measured by forward digit span, was the only measure that was important for reading accuracy and fluency but not mathematics achievement, while passive verbal memory contributed to oral reading fluency but not reading accuracy. While these findings are generally consistent with many previous studies (Swanson et al., 2009b; Peng et al., 2018), they also provide nuance. The critical difference is that the use of verbal memory strategies (e.g., rehearsal) is possible with the digit span but not the verbal memory task. In other words, the ability to engage in top-down manipulation of verbal material was relatively more important for oral reading accuracy than was passive short-term retention of words, while the passive retention of verbal information also contributed to fluency.

Finally, our *post hoc* analyses of individual differences in in-class attentive behavior is unique (to the best of our knowledge) to this study and suggests that a combination of cognitive ability and academic attitudes contribute to engagement in middle-school classrooms. One possibility is that lower-ability students find academic learning more difficult than their higher-ability peers and over time this leads to less positive academic attitudes and less investment in academic learning. The long-term result would be disengagement in classroom settings and with schooling more generally. If correct, then longitudinal studies should show a cross-grade decline in in-class attentive behavior that is mediated by intelligence and academic attitudes. If so, then interventions associated with improving engagement in the classroom might prove useful for these students.

### Learning Difficulties

As noted, individual differences in achievement are continuous and thus cutoffs for learning difficulties are necessarily arbitrary to some extent (Grigorenko et al., 2020). Nevertheless, on the basis of the relation between various outcomes in adulthood and actual academic competencies at different levels of achievement, the 25th percentile is a reasonable cutoff for identifying adolescents who are at risk for long-term educational and occupational issues (Rivera-Batiz, 1992; Ritchie and Bates, 2013; Richmond-Rakerd et al., 2020).

Students with difficulties in both reading and mathematics are at significantly higher long-term risk than are students with difficulties in only one domain. The focus on these students added to the individual differences analyses by identifying the core factors contributing to group membership. The combination of low-average intelligence (*M* = 90, Table 2) and poor in-class attentive behavior was a potent predictor of whether a student fell into the comorbid learning difficulty or typically achieving group (*D* = 2.44). The use of a typically achieving group with high-average intelligence and mathematics achievement likely inflated the size of the multivariate effect. Nevertheless, in comparison with the overall sample (Table 2), the students with comorbid learning difficulties were still about 1 *SD* below average on both intelligence (*d* = 1.13) and in-class attentive behavior (*d* = 1.17), indicating the combination would remain a substantive discriminator of difficulty status relative to students with average intelligence and achievement.

The identification of intelligence and in-class attentive behavior as contributors to learning difficulties confirms prior results (Geary et al., 2012; Willcutt et al., 2013; Peng et al., 2019). The unique contribution here is the identification of their combined contributions to achievement difficulties. Although the logistic regression indicated that they contributed equally to group membership, the finding that intelligence is a strong predictor of attentive behavior suggests a more dynamic relationship, as noted above.

As with comorbid learning difficulties, the combination of intelligence and in-class attentive behavior also discriminated students with mathematics learning difficulties from their typically achieving peers (Willcutt et al., 2013). The difference was that in-class attentive behavior was a stronger predictor of mathematics difficulties than was intelligence, relative to equal contributions for students with comorbid difficulties. As noted, it is possible that low-average intelligence contributes to student disengagement from and less positive attitudes toward academic learning. In this situation, interventions that incorporate components of self-regulation (Wang et al., 2019), enhance academic attitudes (Sisk et al., 2018), or enhance classroom management strategies (Korpershoek et al., 2016) might be particularly helpful for students with both comorbid and mathematics learning difficulties.

Intelligence also contributed to risk of reading difficulties. However, in contrast to students with comorbid or mathematics difficulties, in-class attentive behavior did not emerge as a core discriminator of these students from their typically achieving peers. Rather, the combination of relatively low visuospatial attention and verbal short-term memory, along with intelligence, discriminated them. The finding for verbal short-term memory (Helland and Asbjørnsen, 2004) and visuospatial attention (Friedrich et al., 1985) is in keeping with prior results and demonstrates that their combined effect is more important than either effect in isolation. The null result for in-class attentive behavior is surprising, given the individual differences results and Willcutt et al.’s (2013) finding of increased rates of attention-deficit disorder among students with reading disability. The differences are likely related to the multiple factors that can disrupt reading achievement and the heterogeneity of reading difficulties groups across studies. The reading difficulty group here appears to have circumscribed deficits in the top-down control of visual attention and in verbal short-term memory.

### Limitations

The correlational nature of the data is the primary limitation and precludes strong causal statements. We assessed a broader array of domain-general cognitive and non-cognitive predictors of reading and mathematics achievement than is typical for this type of study, but this does not preclude the contributions of other factors that we did not assess. The inclusion of other factors, such as the contributions of the home environment and other known predictors of academic achievement (e.g., rapid automatic naming), might change the relative importance of the factors that we identified, although this remains to be determined. Moreover, the emergence of in-class attentive behavior as a predictor of academic outcomes should not be interpreted as an indicator of attentional deficits, as the participants’ classroom behavior could simply reflect disengagement from schooling rather than attentional deficits *per se*. Despite these limitations, our broad assessments and analytic approaches enabled a thorough evaluation of the cognitive and non-cognitive factors that are common to mathematics and reading achievement and learning difficulties, as well as factors that disproportionately contribute to achievement in one domain or the other. The combination has implications for the identification of at-risk students and for the development of interventions to reduce these risks.

## Data Availability Statement

The data and R code used in these analyses are available in Open Science Framework, https://osf.io/cuvs5/.

## Ethics Statement

The study was reviewed and approved by the University of Missouri Institutional Review Board (IRB; Project 2002634, “Algebraic Learning and Cognition”). Written informed consent to participate in this study was provided by the participants’ legal guardian/next of kin.

## Author Contributions

DG drafted the manuscript and completed most of the analyses. JS completed some analyses. MH and LN managed data collection and quality control. All authors contributed to the writing of the final manuscript.

## Conflict of Interest

The authors declare that the research was conducted in the absence of any commercial or financial relationships that could be construed as a potential conflict of interest.

## Footnotes

1 https://nces.ed.gov/nationsreportcard/

2 https://www.millisecond.com

3 https://www.qualtrics.com

4 https://osf.io/qwfk6/

5 http://garp.education.uci.edu/msalt.html
